# Simple eco-friendly synthesis of the surfactant free SnS nanocrystal toward the photoelectrochemical cell application

**DOI:** 10.1038/s41598-017-16445-8

**Published:** 2017-11-28

**Authors:** Xiaoguang Huang, Heechul Woo, Peinian Wu, Hyo Jin Hong, Wan Gil Jung, Bong-Joong Kim, Jean-Charles Vanel, Jin Woo Choi

**Affiliations:** 1 State Key Laboratory of Advanced Technology for Materials Synthesis and Processing, Wuhan University of Technology, Wuhan, 430070 People’s Republic of China; 2 Advanced Photonics Research Institute, Gwangju Institute of Science and Technology, 1 Oryong-dong Buk-gu, Gwangju, 500-712 Korea; 3 School of Materials Science and Engineering, Gwangju Institute of Science and Technology, Gwangju, 61005 Republic of Korea; 4 Laboratoire de Physique des Interfaces et des Couches Minces, LPICM, UMR 7647 CNRS, Ecole polytechnique, Route de Saclay, 91128 Palaiseau Cedex, France

## Abstract

A simple, low cost, non-toxic and eco-friendly pathway for synthesizing efficient sunlight-driven tin sulfide photocatalyst was studied. SnS nanocrystals were prepared by using mechanical method. The bulk SnS was obtained by evaporation of SnS nanocrystal solution. The synthesized samples were characterized by using XRD, SEM, TEM, UV-*vis*, and Raman analyses. Well crystallized SnS nanocrystals were verified and the electrochemical characterization was also performed under visible light irradiation. The SnS nanocrystals have shown remarkable photocurrent density of 7.6 mA cm^−2^ under 100 mW cm^−2^ which is about 10 times larger than that of the bulk SnS under notably stable operation conditions. Furthermore, the SnS nanocrystals presented higher stability than the bulk form. The IPCE(Incident photon to current conversion efficiency) of 9.3% at 420 nm was obtained for SnS nanocrystal photoanode which is strikingly higher than that of bulk SnS, 0.78%. This work suggests that the enhancement of reacting area by using SnS nanocrystal absorbers could give rise to the improvement of photoelectrochemical cell efficiency.

## Introduction

Hydrogen is a non-polluting, efficient and renewable energy carrier, which has been strongly chased to mitigate the global issue, like environmental deterioration and increasing energy scarcity. Among the various methods to fabricate the hydrogen, water splitting is one of the most promising methods because it utilizes solar energy which is clean, abundant, inexpensive compared to many other renewable energy sources^1,2^. The efficient and low-cost production of sustainable hydrogen from sunlight and water for an ideal long-term solution of renewable-energy conversion has motivated an intense research. Photoelectrochemical (PEC) cell is an attractive approach to that aim. There are two photoelectrodes in PEC device structure; the photoanode for the oxygen evolution reaction (OER) and the photocathode for the hydrogen evolution reaction (HER). Until now, most of the PEC cells are built based on multi-composite photovoltaic modules, which contain rare/toxic materials with high processing costs^3–7^. Even though Cd containing photoanodes such as Cd/CdO/CdS heterojunction photoanode^7^, CdIn_2_S_4_/CdS photoanode^3^ and CdS/Zr:Fe_2_O_3_ photoanodes^5^ have shown outstanding and long-term photoelectrochemical activity for water splitting, the high degree of toxicity of Cd is of public health significance. It is well known that even at lower levels of exposure to Cd can cause multiple organ damages.

**Figure 1:**
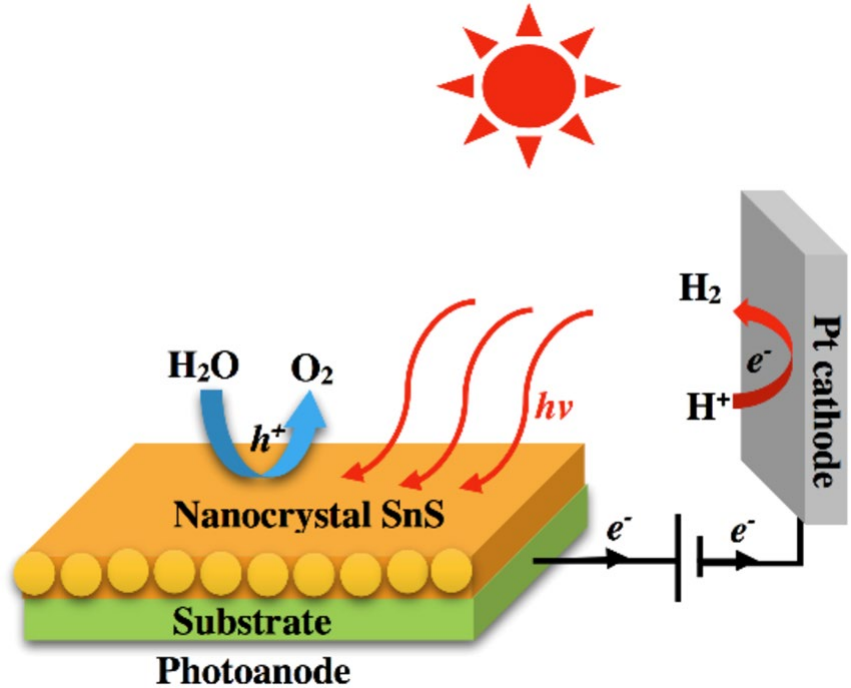
Architecture of the SnS nanocrystals photoanode used in this study.

To this end, numerous works to find earth abundant alternatives have been reported in the past decade^8,9^. As such alternaives, nanostructured earth abundant materials have also been studied and reported. These materials have an enormous potential in energy conversion, mainly because of their high surface to volume ratio which provides increased photon collection area and lowers carrier recombination. Among these nanostructured materials, TiO_2_^10,11^ or ZnO^12,13^ have received most attention in view of their nontoxicity and low cost. However, due to their wide band gap (TiO_2_ ~ 3.2 eV, ZnO ~3.37 eV) they can only be active in the ultraviolet light zone. Therefore, the device based on the TiO_2_ or ZnO could harvest lesser than 5% of the sunlight. On the other hands, WO_3_^14,15^, BiVO_4_^16^ and *α*-Fe_2_O_3_^17,18^ are very attractive materials which can serve as oxygen evolving anodes for absorbing energy from the ultraviolet light zone of solar spectrum because of their band gaps of 2.2, 2.45 and 2.6 eV, respectively. However, there are still some limitations on their performance, such as low absorbance in visible light zone, poor charge-carrier transport, poor collection of photogenerated electrons, and limited chemical stability in an electrolyte under illumination. Recently, tin (II) sulfide (SnS) has emerged as an attractive candidate for low cost catalytic materials of photovoltaic absorbers as well as photoelectrodes for solar water splitting because of their promising high electron (2.37 × 10^4^ cm^2^ V^−1^ s^−1^) and holes (7.35 × 10^4^ cm^2^ V^−1^ s^−1^) mobilities. Genernally, it is well knwon that the high carrier mobility of semiconductor is preferable for photovoltaic device applications since it prevents photogenerated excitons from recombination. Therefore, semiconductor with low carrier mobility greatly hinders its practical applications^19^. For this reason, high carrier mobility of SnS at the photoanode surface is preferable because it can promote fast photocatalytic process^20,21^. To take advantages of this kind of SnS nanocrystals, an ideal architecture of nanostructured films could be conceived, consists of SnS nanocrystals with high crystallinity and good electrical property on the substrate (Fig. 1).

SnS is known as a potential candidate for solar energy application due to its excellent optical and fascinating electrical properties^22,23^. The optical anisotropy of SnS showed an antireflection properties which results in high photoactivity. There is only a weak interaction in the 2D nature of SnS. This interaction allows easy separation and fabrication of layered composite structures. The layered composite structures create a wide range of Van der Waals heterostructures. Therefore, the processability of SnS makes it a perfect system in exploring new 2D feature^24^. Up to date, SnS nano sheets have been synthesized using various methods, for example: chemical bath deposition^25^, chemical vapor transport^26^, chemical vapor deposition (CVD)^27^, colloidal nanoparticle synthesis^28–31^, electrochemical deposition^32^, vacuum evaporation^33^, spray pyrolysis^34^, and dip deposition^35^. Among these SnS layers, the mono-layer nanostructured SnS photoanode has exhibited the high photocurrent density of 7 mA cm^−2^, which was synthesized by chemical spray pyrolysis^36^. However, this method used thiourea, which is environmentally hazardous, and is a toxic material as well. SnS layer fabricated with nanocrystalized SnS could be a promising alternative to traditional solution-based methods, which does not require high vacuum and high process temperature^37^. However, conventional nanocrystals contain surface ligands which act as electrical insulator in PEC device. Therefore, surfactant free stable nanocrystals are highly desirable. Here, we present a mechanical alloying (MA) process which is a simple, low cost, and eco-friendly pathway to produce surface clean semiconductor nanocrystals of surfactant free tin sulfide (SnS). To the best of our knowledge, the photocurrents in this work are higher than the any of previously reported SnS systems.

## Results and Discussion

### Structural and Morphological Characterization

**Figure 2:**
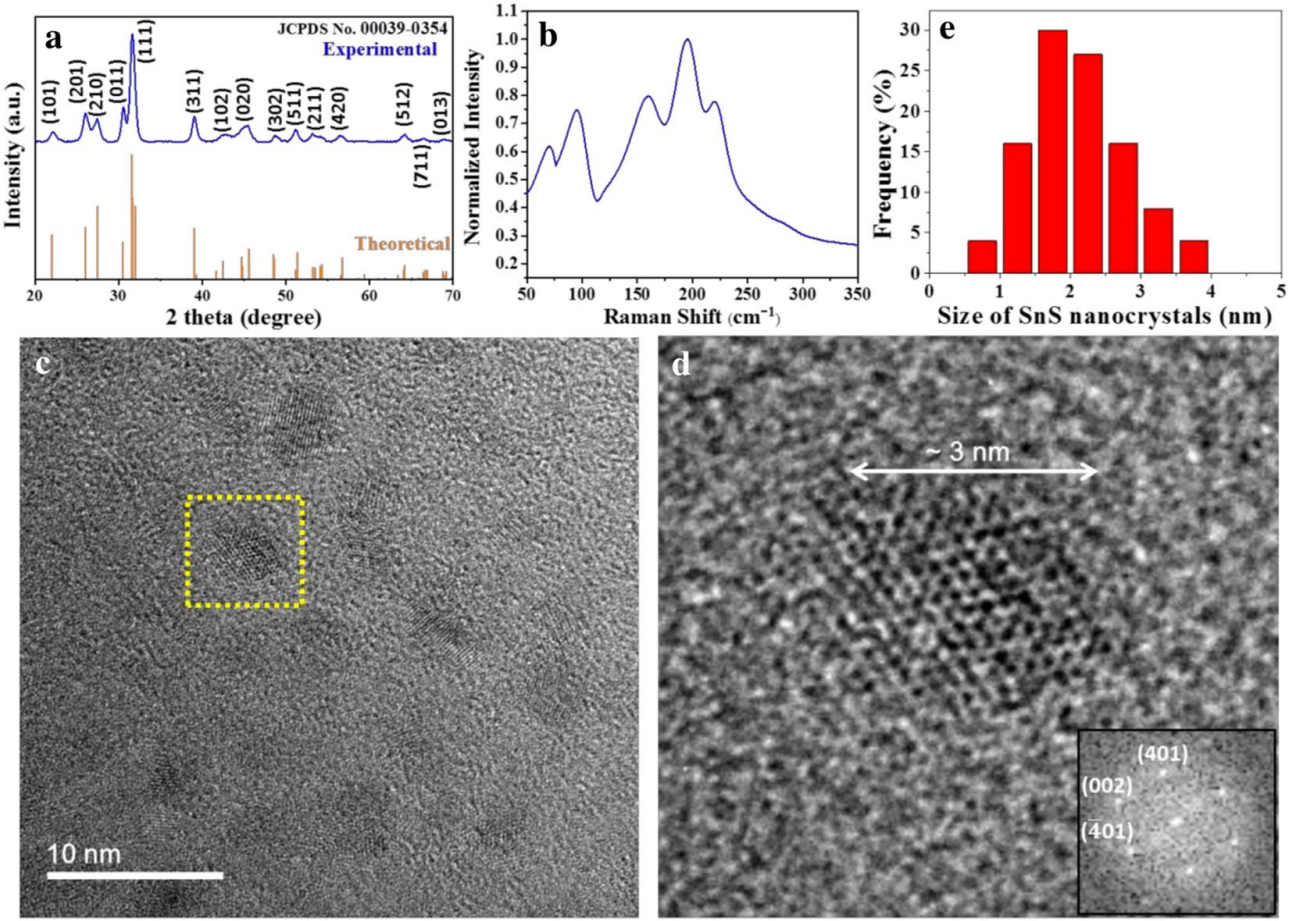
(**a**) X-ray diffraction pattern of the SnS nanocrystals. (**b**) Raman spectra of the SnS nanocrystal. (**c**) Bright-field TEM image of SnS nanocrystals. (**d**) The high resolution TEM image (HRTEM) of SnS nanocrystal from the boxed region in figure (**c**) with the corresponding fast furrier transformation (FFT) pattern. (**e**) Statistical size distribution of the SnS nanocrystals.

Powder X-ray diffraction (XRD) data was used to confirm the SnS phase purity. Figure 2a represents the XRD pattern of the SnS nanocrystals particles. Every peak in Fig. 2a was well indexed with values in the standard card^38^ of the orthorhombic SnS phase (JCPDS No. 00039-0354), the structure with lattice constants (a 1⁄4 4.329 Å, b 1⁄4 11.190 Å, c 1⁄4 3.983 Å). In addition, the diffraction peaks were strong and sharp, which suggest that the SnS were well crystallized. And the result also confirmed the presence of highly pure SnS nanocrystals phase without any impurity.

The use of Raman spectroscopy to probe the detailed structure of materials is reviewed comprehensively. Figure 2b shows the Raman spectra of SnS nanocrystal. The Raman modes are observed at 70, 95, 160, 195, 219 cm^−1^ corresponding to one B_1g_ or B_2g_ mode, one A_g_, one B_3g_ (LO) and two A_g_ (LO), modes respectively. They are in good agreement with what we can find from the literatures. It confirms the phase purity of SnS nanocrystal, while no impurities, such as SnS_2_ (315 cm^−1^), Sn_2_S_3_ (153 cm^−1^) and SnO_2_ (472 cm^−1^) were observed.

We observed the thickness of SnS nanocrystal thin film about 180 nm in cross-section view of SnS nanocrystal thin film (Fig. S1). The morphology of SnS nanocrystals were shown in Fig. 2c. It can be observed that the SnS nanocrystals were distributed homogeneously. TEM analysis (Fig. 2d) roughly showed isolated SnS nanocrystals. Lattice fringes are displayed in the image, indicating that the SnS nanocrystals were well crystallized. The statistical results of size distribution for the SnS nanocrystals are presented in Fig. 2e. Columns in the Fig. 2e represents the statistical count ratio corresponding to the grain size. The grain size of the individual nanocrystals was found from TEM images. The summarized datum of the individual nanocrystal size could be used to calculate statistical size distribution. It can be roughly estimated that the grain size is between 1.5–2.5 nm, suggesting that the SnS nanocrystals are homogeneous.

**Figure 3:**
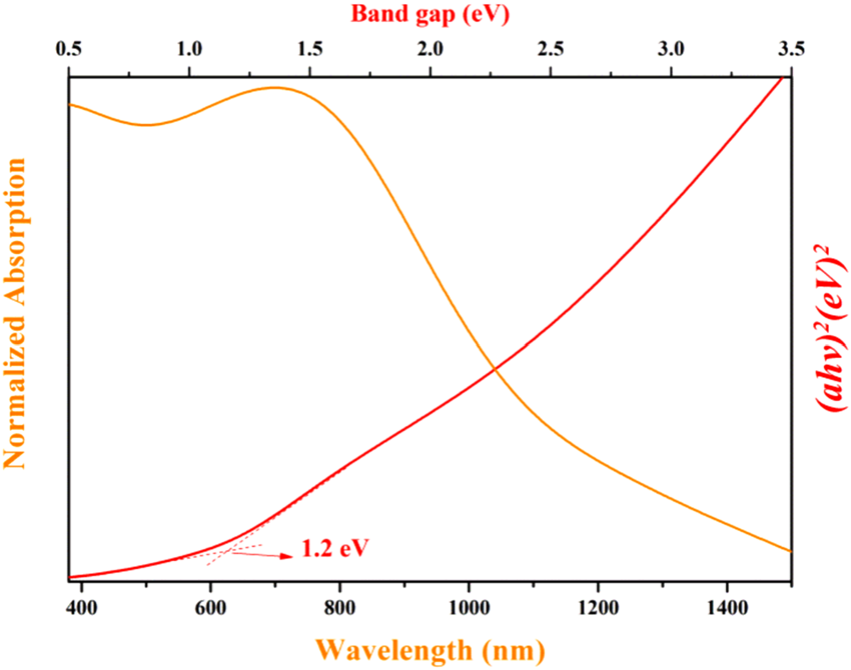
UV-Vis absorption spectrum of the SnS nanocrystals.

Since a true optical band gap is an indication of a pure phase of a material, we performed UV-Vis absorption measurements using an integrating sphere and determined the band gap of our synthesized SnS nanocrystals. The results are shown in Fig. 3. The spectrum covered almost the entire visible range, suggesting SnS nanocrystals can absorb energy from the ultraviolet light zone of solar spectrum. A direct band gap value of 1.20 eV was characterized from the extrapolated intercept with the energy (*hv*) axis, which is well in accordance with literature values and with that of standard commercial bulk SnS as well. This indicates that our synthesized SnS nanocrystals are in pure crystal phase. However, we could not observe the possible blue shift in the band gap due to the larger size of the particles.

### The SnS nanocrystals Photoanode Performance

The thickness dependence of the SnS films on the catalytic activity was then investigated (Fig. S2). We first describe the results obtained from the SnS nanocrystal samples. Low current density was observed for the thin (50 ± 20 nm) films while thicker films (100 ± 30, 200 ± 50 nm) displayed better catalytic currents as their thickness increased. However, the current density decreased when the films thickness reached 300 ± 100 nm. Meanwhile, the films with thicknesses of 200 nm have shown good stability during electrolysis. The similar behavior was observed for the bulk sample.

**Figure 4:**
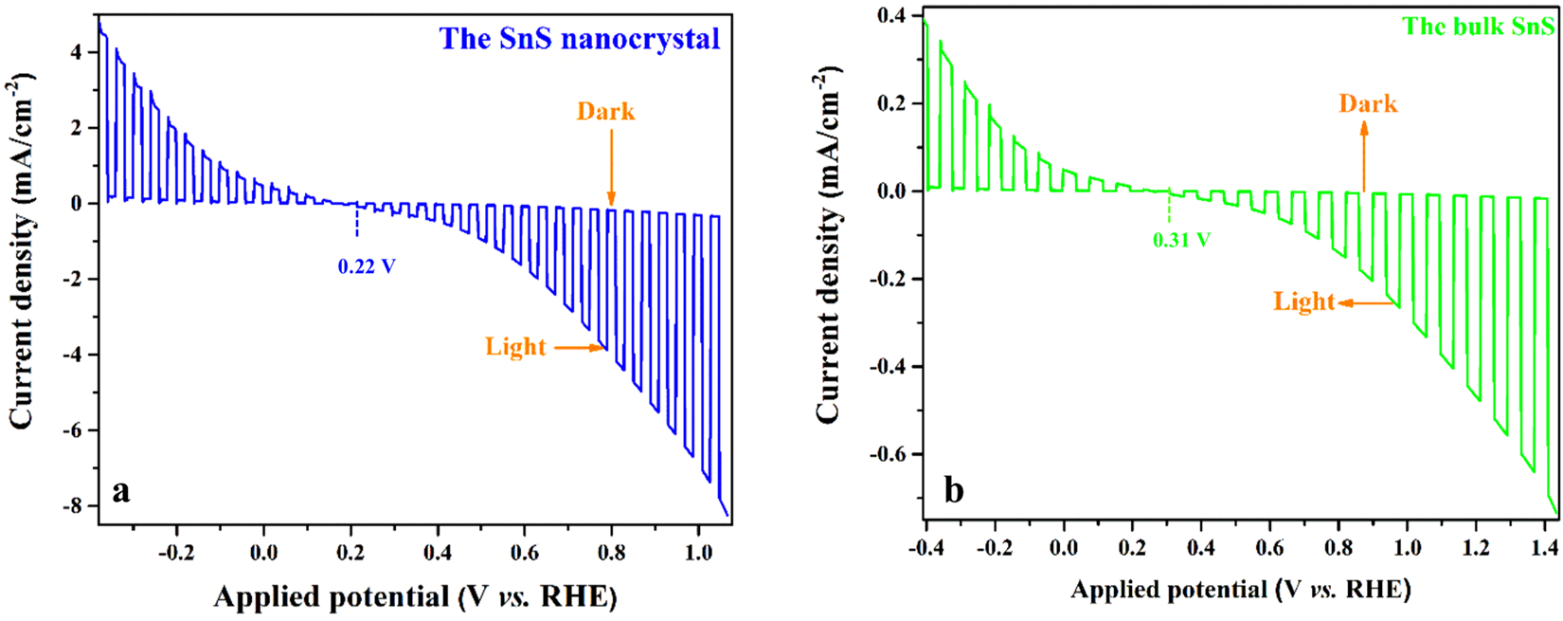
(**a**) Linear sweep voltammogram of the SnS nanocrystal under chopped illumination; (**b**) Linear sweep voltammogram of the bulk SnS under chopped illumination. The working area of the electrode: 1 cm^2^.

The linear sweep voltammogram spectrum of PEC cell recorded at 50 mV s^−1^ in 0.1 M K_4_Fe(CN)_6_ and 0.01 M K_3_Fe(CN)_6_ with chopped visible light is shown in Fig. 4. A cathodic photocurrent could be possible in the n-type semiconductor because of the accumulation of majority carriers on the electrolyte side ascribed to the part below the flat band. The above part as the band starts to align in response to the applied potential is the depletion part. The cell onset potential (V_on_) was found to be 0.22 V versus RHE which was 90 mV more negative than the bulk one. The lower photovoltage may be attributed to the difference in the density of photoanode material, which changes the hole injection barrier. A photocurrent is about 7.6 mA cm^−2^ at 1.05 V versus RHE and it reaches 8.2 mA cm^−2^ at more cathodic potentials. The dark currents of the SnS nanocrystals and the bulk SnS (Fig. 4b) remained at a level of less than 0.8 mA cm^−2^ between 0.4 V and 1.1 V versus RHE. In contrast to the visible-light irradiation, the SnS nanocrystals presented a much enhanced photocurrent density of 7.6 mA cm^−2^ at 1.05 V versus RHE, approximately, 9.3 times larger than that of the bulk material. Increased reaction area with water due to the larger surface area of SnS nanocrystal layer compared to the bulk SnS layer could be the reason of this incredibly improved photocurrent density. It can be noticed that at −0.2 V vs. RHE the spikes flatten out, suggesting that the kinetic barriers have been solved^39^. High photocurrent of the cells is obtained that is comparable with other previously reported SnS systems^30,32,36,37,40–46^ in the literature listed in Table 1.

In a number of reports in Table 1 present a smart strategy to increase the short circuit current for the further improvement of the photoelectrochemical cell. Ray’s group obtained short-circuit current density of 0.3 mA cm^−2^ by optimizing molar concentration ratio of S^2−^/Sn^2+^ ^30^. They found 1.2 mA cm^−2^ of short-circuit current with device based on Cu doped sprayed SnS thin films^36^. After then, nanostructured SnS photoanodes were adopted and the device exhibited a 0.42 mA cm^−2^ of short-circuit current^40^. On the other hands, Xi’s group also reported high photocurrent density 3 mA cm^−2^ by using SnS atomic layer^37^. The cells fabricated in this work have shown a short circuit current density of 0.45 mA cm^−2^ which means higher short-circuit current density is in urgent need for the improvements of mass production and practical applications. And applying atomic layer material in 0.5 M Na_2_SO_4_ would be a good way to improve short-circuit current density.

**Table 1:** Photocurrent density of SnS photoelectrodes in different systems.

System	Light power (mW cm^−2^)	Short-circuit current density (mA cm^−2^)	Ref.
FTO-SnS: 0.1 M K_4_Fe(CN)_6_ + 0.01 M K_3_Fe(CN)_6_	100	0.45	This work
FTO-SnS: 0.1 M Na_2_S_2_O_3_	30	0.3	30
FTO-Cu: SnS: 0.1 M K_4_Fe(CN)_6_ + 0.01 M K_3_Fe(CN)_6_	60	1.2	36
FTO-SnS: 0.1 M K_4_Fe(CN)_6_ + 0.01 M K_3_Fe(CN)_6_	100	0.42	40
ITO-SnS: 0.5 M Na_2_SO_4_	100	3	37
FTO-SnS: 0.1 M Na_2_S_2_O_3_	30	1	41
FTO-SnS: I^3−^/I^−^	100	0.087	42
ITO-SnS-TiO_2_: 0.5 M Na_2_S	100	1.5	43
FTO-SnS: I^3−^/I^−^	100	0.07	44
SnO_2_-SnS: 0.1 M FeCl_3_	100	0.65	47
FTO-SnS: 0.1 M FeCl_3_	100	0.25	48
Mo-SnS: 0.1 M H_2_SO_4_	100	0.01	49
FTO-SnS: 0.1 M Eu(NO_3_)_3_	100	0.017	50

The maximum theoretical photocurrent density (*J*_*ph*, *max*_) for the material with band gap of 1.2 eV was estimated to be 36.7 mA cm^−2^ by calculating from eq. 1 ^51^.

1 $${J}_{ph,\max }={\rm{e}}{\int }_{{\rm{1}}\mathrm{.2}\,\mathrm{eV}}^{\infty }{\rm{\Phi}}\mathrm{dE}={\rm{36.7}}\,{\rm{mA}}\,{{\rm{cm}}}^{-2}\,$$

**Figure 5:**
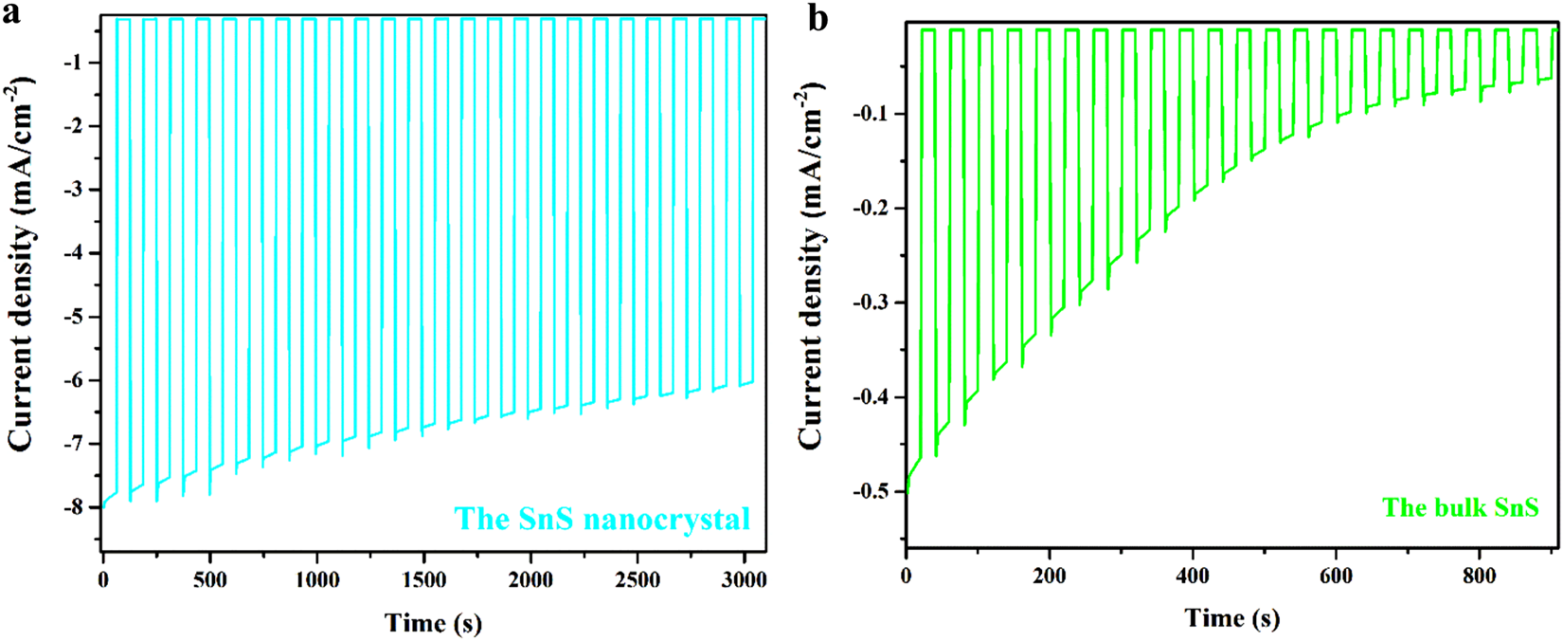
(**a**) Chronoamperometry of the SnS nanocrystal at 1.23 V versus RHE with chopped visible light; (**b**) Chronoamperometry of the bulk SnS at 1.23 V versus RHE with chopped visible light. The working area of the electrode: 1 cm^2^.

Therefore, the saturation current density, *J*_*ph*_ (7.6 mA cm^−2^) in our case reaches only about 20% of the theoretical maximum photocurrent *J*_*ph*,*max*_ (36.7 mA cm^−2^). In other words, it still remains about 85% of possible increase in the photocurrent density that can be achieved by developing pure SnS. One of the origins of this loss could be the nature of surfactant free SnS nanocrystal. Even though surfactant free SnS nanocrystal could provide better conductivity in the device, reaction kinetics at the surface of the SnS nanocrystals could be sluggish due to the surface trap state at the surface. The photocurrent spikes observed in the Fig. 5 indicate the presence of theses surface traps^36^.

The reproducibility of the photocurrent response can be switched from the “ON” state to the “OFF” state by chopped visible light. Stability measurements were then carried out with chopped light at 1.23 V versus RHE. The results are shown in Fig. 5.

It takes around 0.5 s for response and decay in Fig. 5a, suggesting rapid response characteristics. A photocurrent density of 7.5 mA cm^−2^ was decreased by only 24% after 50 min. At the same time, the low dark current ensured the excellent stability of the cell. It is worth noting that the photocurrent densities of the SnS nanocrystals showed high stability even after 3,000 s of irradiation (24% decrease), while the bulk material displayed 87% decrease after 900 s (Fig. 5b). This is a clear evidence for the enhanced stability of the SnS nanocrystals.

SEM images taken before and after the chronoamperometric measurements of nanocrystal SnS are shown in Fig. S1. After the measurements, SEM showed that pieces of SnS have been detached from the surface, probably because of the H_2_ bubbles pulling the catalyst.

**Figure 6:**
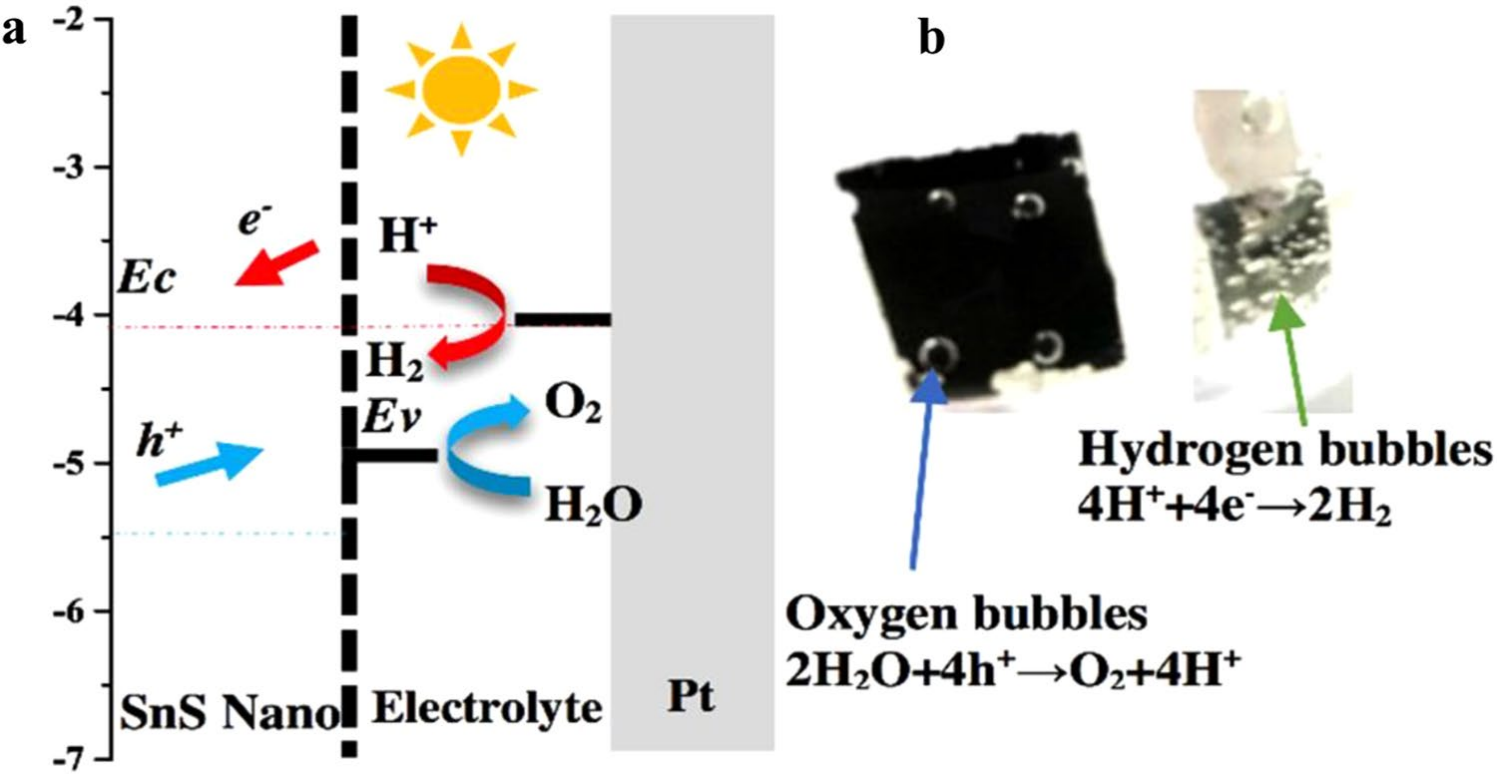
The energy diagram of the PEC cell: the position of the conduction and valence bands of SnS thin film relative to the water oxidation and the water reduction potential (**a**). The digital photograph presented the phenomena of water splitting at 1.2 V versus RHE. (**b**) The working area of the electrode: 1 cm^2^.

Figure 6 described the position of the conduction and valence bands of the SnS nanocrystals edges and the redox levels of the electrolyte. The photogenerated holes were extracted from the band edge of the SnS nanocrystals to the redox levels of the electrolyte. The electrolyte accepted the photo-generated holes from the SnS nanocrystals valence band, and then the oxygen produced at the photoanode surface. Meanwhile, the Pt-counter electrode received photogenerated electrons from the conduction band through the external wire and the hydrogen came out at the Pt surface. The visual phenomena of the oxygen and hydrogen evolution reactions are displayed in Fig. 6b.

To quantitatively evaluate its photo-conversion efficiency, incident photon-to-current conversion efficiency (IPCE) measurements were carried out and the results are shown in Fig. S3. The SnS nanocrystal photoanode possessed an IPCE of 9.3% at 420 nm, strikingly higher than the 0.78% efficiency of bulk sample.

The ratio metric power-saved figure-of-merit *Ф*_*saved*, *NPAC*_ (NPAC = non-photoactive, identical catalyst) (eq. 2) can estimate the ability of a photoanode to achieve H_2_ evolution. *Ф*_*saved*, *NPAC*_ is obtained at the maximum power, it is less catalyst- dependent^51^.

2 $$\begin{array}{rcl}{{\Phi}}_{{saved}{,}{NPAC}} & = & {\eta }_{F}\times \frac{| {J}_{{photo},m}| \times [{E}_{{light}}({J}_{{photo},m})-{E}_{{dark}}({J}_{{photo},m})]}{{P}_{{\rm{in}}}}\\  & = & {\eta }_{F}\times \frac{| {J}_{{photo},m}| \times {V}_{{photo},m}}{{P}_{{in}}}\end{array}$$

Where the faradaic efficacy (*η*_*F*_) assume to be 100%, P_in_ represents the power of the incident illumination, and J_photo,m_ and V_photo,m_ stand for the photocurrent and photovoltage at the maximum power point respectively. Character “m” represents maximum. *J*_*photo*_ is the photocurrent density which is obtained by the current density under illumination (*J*_*light*_) minus the current density of the corresponding catalyst (*J*_*dark*_). The photovoltage *V*_*photo*_ is given by the difference between the potential applied to the photoanode under illumination (*V*_*light*_) and the potential applied to the catalyst (*V*_*dark*_). As expected, the *Φ*_*saved*,*NPAC*_ values of the SnS nanocrystal photoanode (5%) were significantly higher than that of the bulk SnS system (0.158%).

## Conclusion

In conclusion, the SnS nanocrystals as a H_2_-evolving catalyst yields a novel nanostructure type of photoelectrode in water splitting. This system is based on earth-abundant elements and can be easily processed using low cost and low temperature spray-casting method. A simple, low cost, non-toxic and eco-friendly pathway was used to synthesize sunlight-driven tin sulfide photocatalyst. The SnS nanocrystals were well crystallized and their grain size is between 1.5–2.5 nm. The SnS nanocrystals exhibited a direct optical band gap of 1.20 eV. The linear sweep voltammogram showed that the SnS nanocrystals presented photocurrent density of 7.6 mA cm^−2^ which is dramatically larger than that of bulk SnS and is higher than the any of previously reported SnS systems as well. The stability experiment confirmed that the SnS nanocrystals were more stable than the bulk SnS. The SnS nanocrystal photoanode possessed an IPCE of 9.3% at 420 nm, strikingly higher than the 0.78% efficiency of bulk sample. Moreover, figure-of-merit, *Ф*_*saved*, *NPAC*_ was evaluated and discussed. Their *Ф*_*saved*, *NPAC*_ values of the SnS nanocrystals and the bulk SnS reached 5% and 0.158%, respectively. Based on low cost, low fabrication temperature and other various advantages, this work suggests that the SnS nanocrystal absorbers hold great promise for bringing a wealth of eco-friendly environment.

## Experimental Section

### Measurement characterization

The XRD of SnS nanocrystals were performed by D8 Advance X-ray diffractometer (Rigaku Dmax-RB with Cu Kα X-ray source, Germany). UV-*vis* absorption spectra were characterized by using a Lambda 750 S UV-*vis*-NIR spectrophotometer. The morphology of the SnS nanocrystals were recorded with a field-emission transmission electron microscope (TEM, JEM-2100F, Japan) operated at 200 kV. Electrochemical measurements were investigated using a CHI 660E electrochemical workstation (Chenhua, Shanghai, China). A visible-light source performed by a 350 W xenon lamp equipped with a UV-cut-off filter (providing visible-light with >420 nm). A power meter (model FZ-A) used to detected the incident light intensity (~100 mW cm^−2^). IPCE spectra were operated on a QE/IPCE Measurement Kit (Newport, USA). All the kit components automatically were in control of Oriel Tracq Basic V5.0 software. A 300 W Xe lamp was used as the visible-light source, through a monochromator (74125 Oriel Cornerstone 260 1/4 m) onto the cell, and the monochrometer generated a photocurrent action spectrum with a sampling interval of 10 nm through the spectral range (420–920 nm) and a current sampling time of 2 s, where the light intensity and the generated photocurrent were detected with a 2931-C dual channel power/current meter and a 71675 calibrated UV silicon photodetector. IPCE can be calculated concretely as follows:

3 $${IPCE}\,=\,\frac{{hcl}}{\lambda {J}_{{light}}}$$

where *h* is the Planck’s constant, *c* is the speed of light, *I* is the measured photocurrent density at a specific wavelength, λ is the incident light wavelength, and *J*_*light*_ is the recorded irradiance intensity at a specific wavelength.

## Synthetic Methodology

### Synthesis of the SnS nanocrystals

In a typical procedure, the 15 g mixtures of high purity tin and sulfur powders (molar ratio of 1:1) as the starting materials were sealed with stainless steel balls (2–12 mm) in a stainless steel jar. The mass ratio of ball and powder turn to 10:1. The jar was then loaded on a SFM-3 Mixer/mill machine to start mechanical alloying process. After 30 hours, the synthesis done, the particles were taken out the jar.

### Synthesis of the bulk SnS

A typical dissolution experiment as follow: 325 mg the SnS nanocrystals powder into a three-neck round-bottom flask. Distilled en (5 mL) and edt (0.45 mL) were then added. The mixture was lightly heated under stirring and was sonicated for a 10 min. A heating mixture was then used to further heat the mixture to 50 °C for 15 h with stirring.

## Electrochemical Measurements

### Sample Preparation

The SnS nanocrystals solution was prepared by dispersed 100 mg of SnS nanocrystals in 5 mL of toluene in the glovebox. The solution was stirred at 50 °C for 2 h, and spray casting on a cleaned FTO-coated glass substrate in air. The sample thermal treated in air for 3 h min at 350 °C.

### Photoelectrochemical Cell

The photoelectrochemical (PEC) cell have been built in a three electrode cell attached to a CHI 660E electrochemical workstation. The SnS nanocrystals deposited on the FTO glass as working electrodes, Ag/AgCl electrode (3.5 M KCl) was used as reference electrodes and Pt foil was counter electrodes. Potentials are quoted against the RHE. The cell employed the aqueous solutions of N_2_-saturated 0.1 M K_4_Fe(CN)_6_ and 0.01 M K_3_Fe(CN)_6_ as the electrolyte. Linear sweep voltammetry (LSV) measurements were carried out at 50 mV s^−1^. The stability study (photo corrosion) was performed in the N_2_-saturated 0.1 M K_4_Fe(CN)_6_ and 0.01 M K_3_Fe(CN)_6_ electrolysis with SnS photoanode. All the PEC measurements were studied under no stirring and room temperature environment.

## Electronic supplementary material

Supplementary Information
