# Supplementary material for: Simple eco-friendly synthesis of the surfactant free SnS nanocrystal toward the photoelectrochemical cell application
Source: Sci Rep. 2017 Nov 28;7:16531. doi: 10.1038/s41598-017-16445-8 (PMC5705658; doi:10.1038/s41598-017-16445-8)
Supplement: Supplementary file 1 — Supplementary Information [file 41598_2017_16445_MOESM1_ESM.doc]

Simple eco-friendly synthesis of the surfactant free SnS nanocrystal toward the photoelectrochemical cell application

Xiaoguang Huang,* ^a^ Heechul Woo, ^b^ Peinian Wu,^a^ Hyo Jin Hong,^c^ Wan Gil Jung,^c^ Bong-Joong Kim,^c^ Jean-Charles Vanel,^d^ and Jin Woo Choi.* ^b^.

^a^ State Key Laboratory of Advanced Technology for Materials Synthesis and Processing, Wuhan University of Technology, Wuhan 430070, People’s Republic of China.

^b^Advanced Photonics Research Institute, Gwangju Institute of Science and Technology, 1 Oryong-dong Buk-gu, Gwangju 500-712, Korea.

^c^School of Materials Science and Engineering, Gwangju Institute of Science and Technology, Gwangju, 61005, Republic of Korea

^d^Laboratoire de Physique des Interfaces et des Couches Minces, LPICM, UMR 7647 CNRS, Ecole polytechnique, Route de Saclay, 91128 Palaiseau Cedex, France.


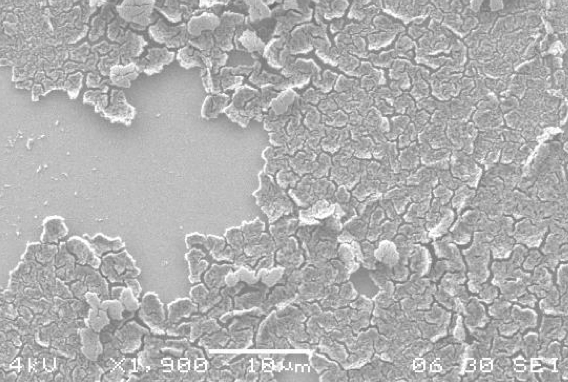

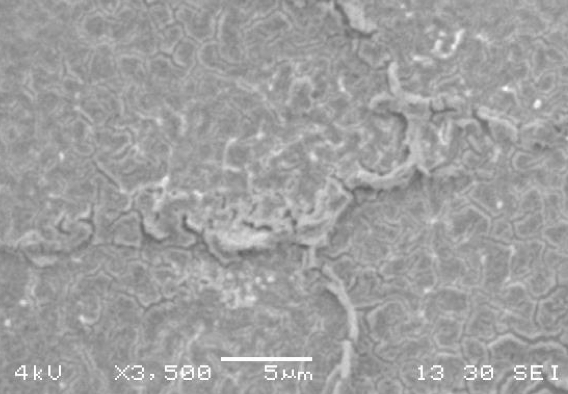

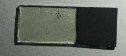

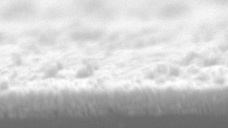


**180 nm SnS**

**100 nm FTO**

**1 cm**

Fig S1. SEM images of SnS nanocrystal film, taken before (left) and after (right) electrolysis.

Insertion: photograph of SnS nanocrystal film with its cross-section view.


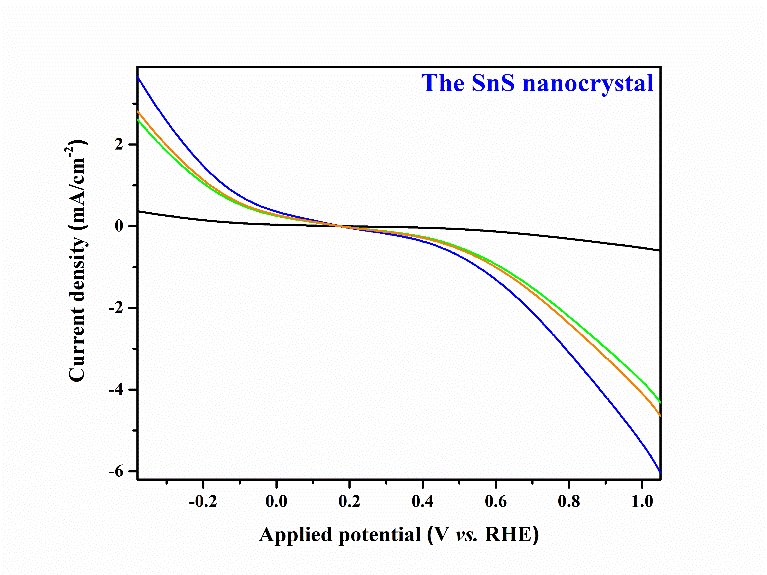


Fig S2. Linear sweep voltammogram of the SnS nanocrystal films, Film thickness: 50±20 nm (black), 100±30 nm (orange), 200±50 nm (blue), 300±100 nm (green). Electrode surface area: 1 cm^2^.


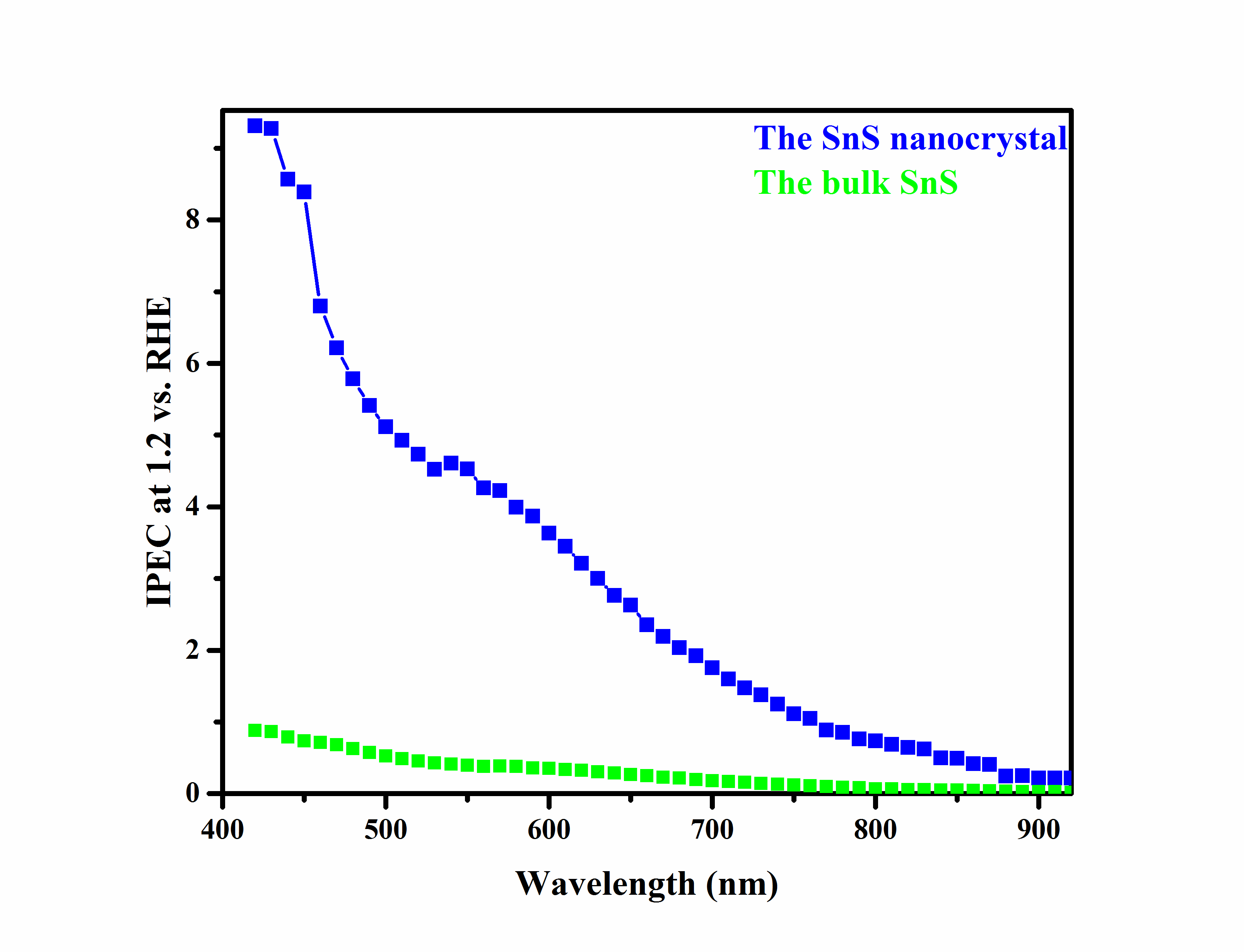


Fig S3. IPCE at 1.2 V vs. RHE
